# Psychological Resilience as a Moderator of the Disability–Mental Health Association in Older Adults

**DOI:** 10.1093/geroni/igaf122.3834

**Published:** 2025-12-31

**Authors:** Sarah Sunghye Kang, Sehyun Baek, Oejin Shin, Soobin Park, Sojung Park, BoRin Kim

**Affiliations:** University of California, Los Angeles, Los Angeles, California, United States; New York University, New York, New York, United States; Illinois State University, Normal, Illinois, United States; Washington University in St. Louis, St. Louis, Missouri, United States; Washington University in St. Louis, St. Louis, Missouri, United States; University of New Hampshire, University of New Hampshire, New Hampshire, United States

## Abstract

Later life is often characterized by disruptive events such as the onset of disability, which can diminish one’s sense of control and purpose. Declines in functional capacity and mobility introduce new stressors that are closely tied to poorer mental health among older adults. Although recent aging research has highlighted the protective role of psychological resilience in responding to major stressors, its role in mitigating symptoms of depression and anxiety among older adults with disabilities remains understudied. Using data from Round 13 of the National Health and Aging Trends Study (n = 7,373), we examined whether psychological resilience moderates the association between physical disability and symptoms of depression and anxiety. Multiple linear regression analyses revealed that greater disability status was significantly associated with higher levels of both depression and anxiety (b = 0.880, p < .001). In contrast, higher psychological resilience was strongly associated with lower levels depression and anxiety (b = −0.131, p < .001). A significant interaction between disability and psychological resilience was identified (b = −0.037, p < .001), indicating a moderation effect. The protective role of psychological resilience on depression and anxiety became more pronounced with increasing disability status. Identifying psychological resilience as a buffer for mental health issues has the potential to inform interventions in aging populations. Moving beyond a deficit-based perspective, these findings support the development of approaches that enhance adaptive capacities and psychological well-being among older adults living with disabilities.

